# TTF1 Expression in Pulmonary Metastatic Rectal Adenocarcinoma

**DOI:** 10.1155/2018/6405125

**Published:** 2018-12-05

**Authors:** Sara Aversa, Cristiana Bellan

**Affiliations:** Department of Medical Biotechnologies, Section of Pathology, University of Siena, 53100 Siena, Italy

## Abstract

Thyroid transcription factor (TTF-1) is a tissue-specific nuclear transcription factor expressed developing thyroid, respiratory epithelium, and diencephalon. TTF-1 is thought to be expressed specifically in most thyroid tumors and in a significant subset of pulmonary neoplasms. However, recent studies on its expression in extrapulmonary carcinomas have demonstrated that TTF-1 is not as specific for lung and thyroid carcinomas as was previously thought: positivity of this marker can be observed, although much less frequently, in some carcinomas arising in other organs, such as the ovaries, endometrium, colon, and breast, as well as in some tumors of the central nervous system. Case reports of patients with TTF-1-positive colon adenocarcinoma are present in medical literature, but there are only a few cases of TTF-1-positive rectal adenocarcinoma. We present the case of a patient with rectal adenocarcinoma with lung metastasis found to be TTF-1-positive on immunohistochemistry. A review of the available literature is also included.

## 1. Introduction

The thyroid transcription factor (TTF-1) is a nuclear protein, part of the Nkx2 gene family. Its expression in normal tissues is restricted to the thyroid and pulmonary epithelium [1, 2]. In lung adenocarcinoma, TTF-1 has been considered a highly sensitive (up to 84% sensitivity) and specific marker (85–100% specificity) for primary lung adenocarcinoma, and it is therefore used as a reliable tool in distinguishing primary lung adenocarcinoma from other malignancies [3]. Nevertheless, in recent years, several studies have highlighted that some cancer arising in other organs, in particular in the intestinal tract, can manifest positivity for this marker [1, 2, 4]. Rare cases of patients with TTF-1-positive rectal adenocarcinoma have been reported. Here, we present a case of rectal cancer with TTF-1-positive lung metastasis which highlights the importance of using additional panels.

## 2. Case Presentation

**Figure 1:**
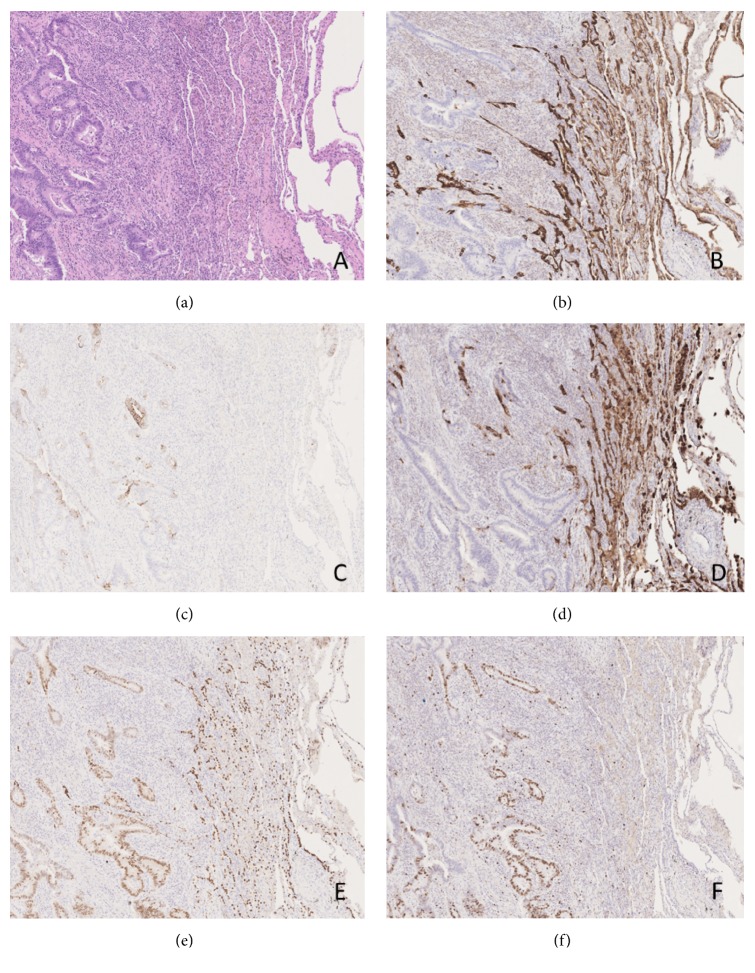
(a) Representative image of the neoplasm (on the left) with normal healthy tissue (on the right) (×200); (b) CK7 immunohistochemical expression (×200); (c) CK20 immunohistochemical expression (×200); (d) Napsin A immunohistochemical expression (×200); (e) TTF-1 immunohistochemical expression (×200); (f) Ki67 immunohistochemical expression (×200).

A 69-year-old patient was diagnosed with a rectal adenocarcinoma (G2) on biopsy after an endoscopic control examination in 2013. He was treated first with radio adjuvant chemotherapy and subsequently with surgery. This combination of treatments has led to a complete response: any residual areas of cancer and lymph node involvement were documented on the surgical piece (yPT1N0 A/I G2 Sec MANDARD). In 2018, during regular oncological follow-up, a subpleural pulmonary nodule in lower lobe of the left lung of about 15x10 mm was detected. Considering the patient's clinical history, his general conditions, and localization of the lesion, a surgical resection of the lung was performed. On the macroscopic exam of the sample, physicians observed a neoformation of 1.9x1.5x0.6 cm, which is whitish, solid, with irregular but well-defined margins, 0.6 cm away from the surgical suture and 0.1 cm from the visceral pleura. Histologic examination demonstrated an epitheliomorphic neoplasm with acinar differentiation (Figure 1). The adenocarcinoma cells were positive for cytokeratin 20 (CK20) and scattered positivity for caudal type homeobox 2 (CDX2) was found. TTF-1 was also strongly and diffusely positive. The tumor cells were negative for CK7 and Napsin A. Retrospective review of his previous primary tumor tissue showed similar histologic findings with TTF-1 positivity. On the basis of the positivity for CK20 and CDX2 with negative CK7 and Napsin A and of the morphology of the lesion, the diagnosis was the following: metastasis from TTF1-positive primary colorectal adenocarcinoma.

## 3. Discussion

Metastasis from CRC adenocarcinoma is a very frequent event that is present in 20% of patients at the time of diagnosis, and an additional 50–60% will develop metastatic disease at the time of progression [5] (Van Cutsem E, Nordlinger B, Adam R, Kohne CH, Pozzo C, Poston G, et al.: Towards a Pan-European Consensus on the Treatment of Patients with Colorectal Liver Metastases; Eur J Cancer 2006;42: 2212–2221). One of the most common sites of CRC metastasis is the lung, and TTF-1 is considered as a highly sensitive and specific marker to distinguish primary lung adenocarcinoma from metastatic adenocarcinoma. However, several studies have highlighted that TTF-1 is not as specific for lung and thyroid carcinomas as was previously thought as it can be found to be expressed, although much less frequently, in some carcinomas arising in other organs and in particular in gastrointestinal neoplasms [1, 4]. In addition, one aspect to consider is some variability in detection depending on the clonal antibody used for testing. There are two clones for TTF-1 known as SPT24 and 8G7G1 [1]. Both clones target the nucleus, but it is proven to be a discrepancy in intensity staining. Since the beginning of its use, spt24 has shown its ability to bring out the cell nucleus with immunohistochemical examination. In their study, Penman et al. have shown how SPT24 has a strong nuclear positivity in colon carcinomas instead of 8G7G1 [6], a result confirmed by other studies carried out on a more extensive case series [7–9]. Colonic carcinomas with nuclear positive for 8G7G1 have been described in literature [10, 11]. What is striking is the colour intensity of the nucleus, which appears to be mild. The advancement of immunohistochemical techniques is unmasking the expression of TTF-1 by neoplasm different from lung and thyroid ones. The set of evaluated data would suggest that SP124 antibody seems to have a higher detection rate than 8G7G1. Nevertheless, an appropriate and prudent approach to case assessment is fundamental. Cases of recurrence with metastatic lesions or occult primary malignancies with pulmonary metastases may constitute a problem of differential diagnosis. Arrangement of TTF-1 with CK20, CDX2, and CK7 is prompted to settle the diagnostic question and the use of SPT24 is suggested for its sensibility. Finally, if available, concurrent evaluation of the primary tumor pathology can be of great help in achieving the correct diagnosis and consequently in offering our patients the most appropriate treatment. It is the set of findings that leads to the most correct diagnosis.

## Data Availability

The authors declare that the data cited in this report in available in the references mentioned, accessed from Pubmed.
